# Identification of the Novel Host Protein Interacting With the Structural Protein VP1 of Chinese Sacbrood Virus by Yeast Two-Hybrid Screening

**DOI:** 10.3389/fmicb.2019.02192

**Published:** 2019-09-26

**Authors:** Xiyan Zhang, Dongliang Fei, Li Sun, Ming Li, YueYu Ma, Chen Wang, Sichao Huang, Mingxiao Ma

**Affiliations:** Institute of Animal Husbandry Veterinary, Jinzhou Medical University, Jinzhou, China

**Keywords:** Chinese sacbrood virus, *VP1*, heat shock protein 70 cognate 5, yeast two-hybrid screening, glutathione S-transferase pull-down, co-immunoprecipitation

## Abstract

Chinese sacbrood virus (CSBV) is the major cause and lead to the collapse of *Apis cerana* colonies. *VP1*, the structural protein of CSBV, shows the highest variation in the amino acid sequences among proteins from different CSBV strains as well as exhibits excellent immunogenicity. However, its function with host protein still remains unclear. To clarify its function with host protein, we screened out host cellular proteins that interact with *VP1* using the membrane protein yeast two-hybrid system. In addition, we verified interactions between heat shock protein 70 cognate 5 (*Hsp70-c5*) and *VP1* using glutathione S-transferase (GST) pull-down and co-immunoprecipitation assays. *VP1* and *Hsp70-c5* were colocalized in the cytoplasm and nucleus. Using western blot and real-time polymerase chain reaction (PCR), *Hsp70-c5* expression in CSBV-infected larvae was upregulated compared with that in healthy larvae. We observed that when we silenced *Hsp70-c5*, *VP1* expression was significantly downregulated. These results demonstrate that *Hsp70-c5* is involved in at least one stage(s) of the viral life cycle.

## Introduction

*Apis mellifera (Am)* and *Apis cerana (Ac)* are major honey bee species in the global beekeeping industry (Zhang et al., 2014). They are heavily infected by different vital viruses (Chen et al., 2004). Among honeybee viruses, Chinese sacbrood virus (CSBV) is the most serious threat to bee health and has caused wide spread concern among beekeepers and researchers (Sun et al., 2018). CSBV usually infects 2-day-old larvae and prevents them from pupating, ultimately leading to their death. The first CSBV inflection was reported in 1972 in *A. cerana* in Fogang and Conghua, Guangdong province, China (Zhang et al., 2002; Sun et al., 2017). In 2008, epidemic outbreaks of CSD caused the death of individual bees and decimated entire bee colonies in Liaoning Province (Mingxiao et al., 2011). Currently, the virus has been frequently reported in numerous regions in China and has had devastating impacts on apiculture.

Chinese sacbrood virus is a typical positive-stranded small RNA virus with a large open reading frame encoding a polypeptide consisting of structural proteins (*VP1*, *VP2*, *VP3*, and *VP4*) and non-structural proteins (Fei et al., 2015). The *VP1* gene has the highest amino acid sequence variation among different CSBV strains and contains the common and major epitope of CSBV (Cheng et al., 2011). Fei et al. (2015) reported that the structural proteins can induce high antibody levels and enhance lymphocyte proliferation, of which *VP1* has good immunogenicity. CSBV is classified into four subgroups based on the *VP1* sequences (Mingxiao et al., 2013). The existence of different CSBV subgroups could be associated with regional variations (Fiil et al., 2008; Hu et al., 2016). Despite the classification of CSBV into four subgroups based on the *VP1* sequences, these subgroups do not exhibit substantial differences with respect to physical and chemical properties, pathogenicity, and immunogenicity (Sato et al., 1994). However, host–cell interactions with partner proteins and the functions of *VP1* remain unknown.

Currently, the analytical methods for evaluation of protein–protein interactions mainly include assays such as co-immunoprecipitation (Co-IP), yeast two-hybridization (Y2H), molecular fluorescence complementation, and glutathione S-transferase (GST) pull-down as well as phage display technology (Vaughan et al., 1996; Stagljar et al., 1998; Ozalp et al., 2005; Rajagopala and Uetz, 2011; Luo et al., 2014). Among these, the Y2H system is highly efficient and is used in *in vivo* screening for protein–protein interactions (Johnsson and Varshavsky, 1994) However, it has some key limitations. For example, the traditional Y2H system is unable to analyze interactions of proteins that cannot access the nucleus such as transmembrane proteins. To address the limitations of existing analytical methods used for the evaluation of transmembrane protein interactions, a Y2H system based on ubiquitin reconstitution has been proposed (Schaafhausen et al., 2011). The advantage of a split-ubiquitin Y2H system is that both bait and prey plasmids adopt strong promoter and high copy number plasmids, which increase protein expression and facilitate the screening of proteins with weak interactions (Wu et al., 2018).

To study the functions of *VP1*, we have screened out host cellular proteins that interact with *VP1* using the membrane protein Y2H system. The results indicated that host heat shock protein 70 cognate 5 (*Hsp70-c5)* interacts with *VP1*. GST pull-down and Co-IP assays were used to verify interactions between *Hsp70-c5* and *VP1*, and they were colocalized mainly in the nucleus and cytoplasm. We report here that *Hsp70-c5* expression in CSBV-infected larvae was significantly upregulated compared with that in healthy larvae. Han et al. (2013) also found *Hsp70* was significantly up-regulated in honeybee larvae challenged by the CSBV. When we silenced *Hsp70-c5*, *VP1* expression was significantly downregulated, demonstrating that *Hsp70-c5* is involved in at least one stage(s) of the viral life cycle. The results provide insights that could facilitate further investigations on CSBV pathogenesis.

## Materials and Methods

### Plasmids, Virus, cDNA Library, and Main Reagents

Plasmids pBT3STE, pET32a, pTT5, pTT5-VP1-His, pEGFP-C2, and pGEX-6P-1-VP1 were provided by the Laboratory of Life Sciences Research Institute of Jinzhou Medical University. The CSBV *VP1* gene, 2–3-day-old *A. cerana* larvae, NMY32 yeast, and *A. cerana* larvae yeast cDNA libraries were constructed in our laboratory. We purchased *Escherichia coli* DH5α and BL21 from TransGen Biotech (Beijing, China) and GST-tagged Protein Purification Kit from Sangon Biotech (Shanghai, China).

### Construction and Evaluation of the CDNA Library

Total RNA from bee larvae was isolated using Trizol reagent (Invitrogen, CA, United States) according to the manufacturer’s instructions. The integrity of the total RNA was analyzed via 1% agarose gel electrophoresis. The concentration and purity of the total RNA were determined by measuring the absorbance at 260 and 280 nm using a spectrophotometer (Eppendorf AG, Hamburg, Germany). mRNA was then isolated from the purified samples using the Oligotex mRNA Kit (Qiagen), and used for constructing the Y2H cDNA library. Briefly, the purified mRNA sample was used for double-stranded cDNA (dscDNA) synthesis. The purified dscDNAs were ligated into a pPR3-N vector (Clontech Mountain View, CA, United States) and then electrotransformed into *E. coli*. The quality of the library was calculated as follows: CFU (colony-forming units)/mL = number of colonies on the plate/10 μL × dilution factor × 1 × 10^–3^ μL. Therefore, total CFU of the library = CFU/mL × total volume of the library liquid (mL). Single bacterial colonies were picked for PCR, and the PCR products were analyzed using 1% agarose gel electrophoresis.

### Construction of pBT3STE-VP1 Bait Plasmid and Plasmid

Total RNA was extracted from purified CSBV and 2–3-day-old *A. cerana* larvae using Trizol reagent according to the manufacturer’s instructions (Invitrogen, CA, United States). RNA was reverse-transcribed into cDNA using a first-strand cDNA synthesis kit (TransGen, Beijing, China). Three pairs of primers were designed for pBT3STE-VP1, pET32a-Hsp70-c5, pEGFP-C2-Hsp70-c5-Flag, and pTT5-Hsp70-c5-Flag. Restriction enzyme sites were inserted based on the CSBV *VP1* sequence (GenBank access No. HM237361.1) and the *Hsp70* gene (GenBank access No. MH122655.1) (Table 1). PCR cycling conditions were as follows: initial denaturation at 94°C for 2 min followed by 30 cycles at 94°C for 45 s, 58°C for 45 s (pBT3STE-VP1) or 55°C for 45 s (pET32a-Hsp70-c5, pEGFP-C2-Hsp70-c5-Flag, and pTT5-Hsp70-c5-Flag), and 72°C for 45 s, and a final extension step at 72°C for 10 min. The PCR product was purified and double digested, following which, the plasmid was extracted and inserted into the vector using the designed restriction site. Finally, the plasmid was transformed into *E. coli* DH5α and identified by endonuclease cleavage and sequencing (Synbio Technologies, Suzhou, China).

**TABLE 1:** Synthetic oligonucleotides for plasmid construction of pET32a-Hsp70-c5, pBT3STE-VP1, pEGFP-C2-Hsp70-c5, pTT5-Hsp70-c5 -Flag and siRNA.

**Plasmid**	**Primers**	**Sequence (5′ to 3′)**	**Restriction enzyme sites**
pBT3STE-VP1	*VP1* F	5′-gcggccattacggccATGGATAAACCGAAGGATATAAGTA-3′	Sif I
	*VP1* R	5′-gcggccgaggcggccTGTACGCGCGGTAAATAAAC-3′	Sif I
pET32a-Hsp70-c5	*Hsp70-c5* F	5′- GCccatggctGCTAAAGCACCTGCAGTT-3′	NcoI
	*Hsp70-c5* R	5′-GCaagcttTTAATACAATTTTGTGAC-3′	HindIII
pTT5-Hsp70-c5 -Flag	Hsp70-c5 F	5′-GCgaattcGCCGCCACCATGGCCAAAGCTCCT-3′	EcoRI
	Hsp70-c5 R	5′-GCaagcttTCATCACTTATCGTCGTCATCCTTGTAATCAGAGCCCTTATCGTCGTCATCCTTGTA -3′	HindIII
pEGFP-C2-Hsp70-c5	*Hsp70-c5* F	5′-GCgaattcGCCGCCACCATGGCCAAAGCTCCT-3′	EcoRI
	*Hsp70-c5* R	5′-GCggtaccTCATCACTTATCGTCGTCATCCTTGTAATCAGAGCCCTTATCGTCGTCATCCTTGTA-3′	KpnI
Hsp70-c5	*Hsp70-c5 F*	5′-GGCGGCGAAGATTTTGATAACC-3′	
	*Hsp70-c5 R*	5′-GCTGGAGGAATGCCGCTGAC-3′	
siRNA 1	siRNA 1	5′-CCTAGCTATGTTGCATTTA-3′	
siRNA2	siRNA2	5′-CACGCGAATTCCTAAGATT-3′	
siRNA 3	siRNA 3	5′-CCAACAAAGCAAACTCAAA-3′	

### Function and Self-Activation Detection of Bait Plasmid

To test bait plasmid with respect to its self-activation and function, it was co-transformed with the control plasmids into the reporter NMY32 yeast strain. Transformants were grown on SD/-Trp/-Leu, SD/-Trp/-Leu/-His, and SD/-Trp/-Leu/-His/-Ala agar plates for 3–5 days (Table 2). The self-activation and function of the bait protein expression plasmid pBT3STE-VP1 was detected based on the growth of the colonies on the plates.

**TABLE 2:** Transfer of various plasmid combinations into the recipient strain NMY32.

**Reaction**	**AD plasmid**	**BD plasmid**	**Coating plate type**	**Remarks**
1	pNubG-Fe65	pTSU2-APP	SD-TL,SD-TLH, SD-TLHA	Positive controls
2	pPR3N	pTSU2-APP	SD-TL, SD-TLH, SD-TLHA	Negative control
3	pPR3N	pBT3STE-WYQ	SD-TL, SD-TLH, SD-TLHA	Self-activation detection
4	pOST1-NubI	pBT3STE-WYQ	SD-TL, SD-TLH, SD-TLHA	Function test
5	–	pBT3STE-WYQ	SD-L	Screen storage reserve

### *Apis cerana* Larvae cDNA Library Screening Using pBT3STE-VP1 as the Bait

After ensuring that our bait was functional in the membrane protein Y2H system by function and self-activation detection, we initiated the screening of host cellular proteins that interacted with *VP1*. We transformed the cDNA library into the NMY32 cells, which contained the appropriate pBT3STE-VP1 decoy plasmid, at 30°C for 3 days. We selected all the potentially positive colonies on SD/-Trp/-Leu and 60 mM 3-amino-1, 2, 4-triazole (3-AT) SD/-Trp/-Leu/-Ade/-His selective media for 1–2 days at 30°C. Yeast colonies cultured on the selective medium could potentially interact with bait and prey proteins. The proteins were recovered from yeast and retransformed into *E. coli* DH5α for subsequent analysis. The proteins were sequenced by Synbio Technologies, Co., Ltd. (Jiangsu, China). The sequences were analyzed using DNSTART biological software (V7.10) and aligned using BLAST in the NCBI/GenBank databases to obtain information on host protein interactions.

### His and *Hsp70-c5* Fusion Protein Expression and Purification From *E*. *coli*

The plasmid pET32a-Hsp70-c5 was transformed into *E. coli* BL21 (DE3). The single selected bacterial colonies were inoculated into 10 mL of Luria-Bertani (LB) medium and incubated at 37°C for 12 h. The cultures (1000 μL) were inoculated into 100 mL LB (100 μg/mL Amp) and cultured at 37°C until the absorbance at 600 nm reached 0.6–0.8. Protein expression was induced by the addition of 0.5 mM isopropyl β-D-thiogalactoside at 28°C for 8 h. The cell precipitates were collected to detect *Hsp70-c5* expression using 8% sodium dodecyl sulfate-polyacrylamide gel electrophoresis (SDS-PAGE) and His-fusion proteins were purified using a His-tagged Proteins Purification Kit (Bio-Works, Sweden).

### GST Pull-Down Assay

The glutathione S-transferase (GST)–VP1 fusion protein was incubated with prepared glutathione agarose beads (Bio-Works, Sweden) at 4°C for 3 h. The beads were incubated overnight at 4°C with the 0.1 mg/mL of input protein His-Hsp70-c5. The agarose complex was eluted, separated, collected, and centrifuged. Finally, the collected protein was analyzed using 8% SDS-PAGE electrophoresis and western blot. As negative control, GST was incubated alone on beads with the *E. coli* lysates.

### Co-immunoprecipitation Assay

The plasmids pTT5-VP1-His and pTT5-Hsp70-c5-Flag were co-transfected into HEK293T cells using Lipofectamine 2000 (GeneCopoeia^TM^, United States). After 24 h of transfection, the cells were collected, treated with radio-immunoprecipitation assay buffer (NaCl, 0.0584 g; Tris–HCL, 100 μL; NP40 25 μL, 0.5%; add water to make up volume to 5 mL) on ice for 30 min. Cellular debris was pelleted by centrifugation (Thermo Scientific Fisher, Waltham, MA, United States) at 12000 rpm at 4°C for 10 min. In all, 500 μL of the cell lysate above was transferred to a 1.5-mL microcentrifuge tube and the cell lysate was incubated with 4 μg of 6^∗^His, His-tagged antibody (Proteintech, Wuhan, China) at 4°C for 1 h. Further, 20 μL of resuspended protein A/G PLUS-Agarose (Santa Cruz Biotechnology, CA, United States) volume was also added. Subsequently, the tubes were capped and incubated at 4°C for 1 h to overnight. Immunoprecipitates were collected by centrifugation at 1500 rpm at 4°C for 3 min. The supernatant was carefully aspirated and discarded. Co-IP products were eluted with 5×SDS loading buffer (Beyotime, Shanghai, China), boiled for 5 min, and identified by western blotting.

### Immunofluorescence Staining and Confocal Microscopy

The plasmids pTT5-VP1-His and pEGFP-C2-Hsp70-c5-Flag were co-transfected into HEK293T cells using Lipofectamine 2000 (GeneCopoeia^TM^, United States). After 24 h of transfection, the cells grown on glass coverslips (Biosorfa, Zhejiang, China) were fixed in 4% paraformaldehyde (Solarbio, Beijing, China) in phosphate-buffered saline (PBS) for 20 min at room temperature. The cells were then washed three times in PBS. The cells were permeabilized with 0.5% Triton X-100 (Sangon Biotech, Shanghai, China) in PBS for 30 min at room temperature and blocked in 2% bovine serum albumin (Solarbio, Beijing, China) in TBST (Tris–HCl, 10 mM; pH, 8.0; NaCl, 150 mM; Tween 20, 0.05%) for 1 h at room temperature. The coverslips were then incubated with 6^∗^His, His-tagged antibody (1:400 dilution) (Proteintech, Wuhan, China) overnight at 4°C. Subsequently, the cells were washed three times in PBS for 5 min. The coverslips were then incubated with secondary antibody and protected from light for 1 h. The secondary antibody was goat anti-mouse antibody (1:2000 dilution) (Abcam, Cambridge, United States). We used 49,69-diamidino-2-phenylindole dihydrochloride (DAPI) (Coolaber, Beijing, China) (1:750 dilution) to stain the cell nuclei and protected from light for 20 min. Finally, the cells were examined using a laser scanning confocal microscope (LEICA, Germany).

### Hsp70-c5 Level After Larvae Infected With CSBV

Three-days-old *A. cerana* larvae were used to this experiment. A total of 20 three-days-old larvae were retrieved from the same colony, following the method of Hu et al. (2016), and randomly distributed into 2 groups, with each group containing 10 larvae. Group I were fed with CSBV at 1.25 × 10^7^ copies/larva (Hu et al., 2016), group II were fed with basic larval diet (BLD) and placed in constant temperature and humidity conditions (T = 34°C, RH = 95%). Each larva was fed 20 μL of a virus suspension mixed with an equal amount of BLD. BLD was used subsequently for daily feeding. The clinical signs in each group of larvae were examined and recorded every day until death of larvae. The dead larvae were detected by RT-PCR for the following viruses during the experiments: BQCV, ABPV, CBPV, DWV, KBV, IAPV, and CSBV. *Hsp70-c5* level after larvae infected with CSBV in this study were analyzed by RT-PCR, the copy number of *Hsp70-c5* is calculated according to the method of Zhou et al. (2017). Drawing a histogram using GraphPad Prism 7.0. Primers for real-time PCR are listed in Table 1. The above assay was performed in triplicate.

We also conducted western blot analysis. Equal amounts of protein samples (12 μg/lane) were separated by 12% SDS-PAGE and then transferred to polyvinylidene fluoride membranes. After blocking, the membranes were incubated overnight at room temperature with anti-Hsp70 (1:1000 dilution). Following three washes, the membranes were further incubated with goat anti-mouse antibody for 1 h. Protein bands were detected using the ECL Western Blotting Substrate (United States). The above assay was performed in triplicate.

### Effect of Silencing *Hsp70-c5* on *VP1* Expression

We performed silencing experiments to investigate the function of *Hsp70-c5* during viral infection. Three pairs of siRNAs of *Hsp70-c5* were designed and synthesized by Guangzhou RiboBio, Co., Ltd. (Table 1). A total of 40 3-days-old larvae were retrieved from the same colony, following the method of Hu et al. (2016), and randomly distributed into 4 groups (groupI-IV), with each group containing 10 larvae. Feeding in constant temperature and humidity conditions (T = 34°C, RH = 95%). After 24 h, group I was fed with BLD containing 1 μg siRNA1, group II was fed with BLD containing 1 μg siRNA2, group III was fed with BLD containing 1 μg siRNA3, group IV was fed BLD alone. 24 h after feeding the siRNA, the diet was replaced with BLD without siRNA and was supplemented daily until the larvae entered the defecation period. After 72 h, three larvae were randomly selected from each group for real-time PCR analysis.

After screening the best siRNA for inhibiting *Hsp70-c5*. A total of 30 three-days-old larvae were retrieved from the same colony, following the method of Hu et al. (2016), and randomly distributed into 3 groups (groupV–VII), with each group containing 10 larvae. Group V larvae were fed with BLD containing 1 μg siRNA, while group VI and group VII were fed with BLD alone. After 24 h, group V was fed BLD containing 1 μg siRNA3 and 1 × 10^7^ copies/μL CSBV (Hu et al., 2016), while group VI was fed BLD with only the same copy number of CSBV. Group VII was fed BLD alone. After 24 h, the diet was switched to BLD without siRNA3 or virus, and was supplemented daily until the larvae entered the defecation period. After 72 h, three larvae were randomly selected from each group for real-time PCR analysis. VP1 expression in larvae was detected using the TransStart Tip Green qPCR SuperMix (TransGen, Beijing, China). The copy number was calculated according to the method of Zhou et al. (2017).

## Results

### Construction of the Y2H cDNA Library

**FIGURE 1:**
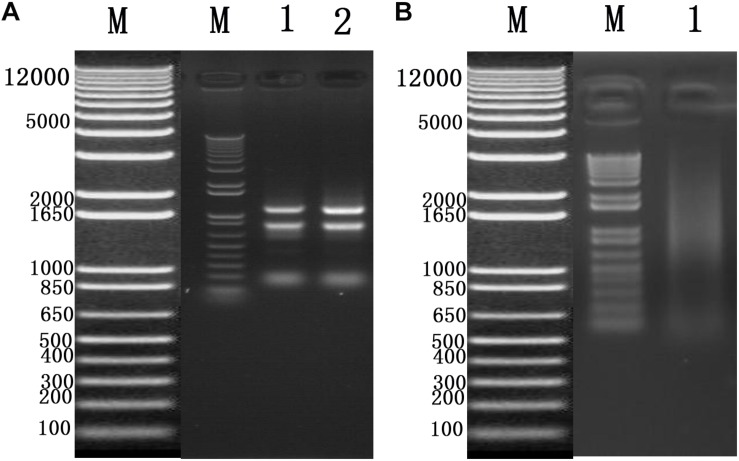
**(A)** Total RNA of Chinese bee larvae: M:DNA Marker DL12000, lane1-2:total RNA of Chinese bee larvae. **(B)** Chinese bee larval mRNA isolation results: M:DNA Marker DL12000, lane1:Chinese bee larval mRNA.

**FIGURE 2:**
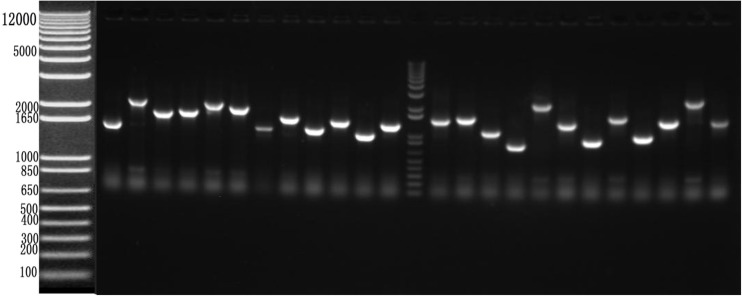
Recombination rate and insert fragment identification of membrane protein yeast cDNA library of *Apis cerana* cerana.

To identify cellular factors that potentially interact with CSBV *VP1*, we needed to construct and evaluate a cDNA library. The integrity of the total RNA extracted using Trizol reagent was assessed via 1% agarose gel electrophoresis, which revealed three bands corresponding to ribosomal 28S, 18S, and 5S RNA; there was no visible degradation (Figure 1A). The OD_260_/OD_280_ ratio of total RNA was 1.95, with a concentration of 139.4 ng/μL, indicating that the total RNA had sufficient purity and quality to construct the cDNA library. After mRNA isolation, the total amount of mRNA was determined to be 5.6 μg. The results of electrophoretic detection are shown in Figure 1B; the mRNA bands were clear, distributed in a diffuse manner, and the bands were evenly distributed, fully meeting the needs for library construction. The library titer was 3 × 10^6^ CFU/mL. The total storage capacity of the original bacterial solution after conversion of 5 mL was 1.5 × 10^7^ CFU. The results of PCR revealed that most inserts were greater than 1200 bp, and the average length was about 1500 bp. None of the electrophoresis lanes running the 24 monoclonal PCR products were blank; therefore the recombination rate of the library was 100%, and the library index was qualified (Figure 2).

### Construction of pBT3STE-VP1 Bait Plasmid

**FIGURE 3:**
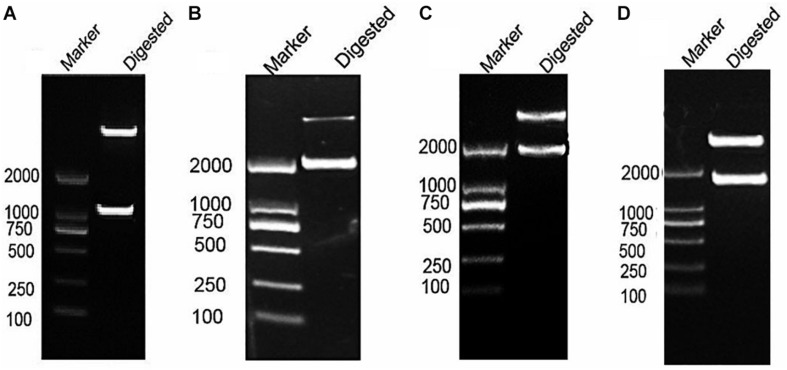
Construction of pBT3STE-VP1, pET32a-Hsp70-C5 and pTT5-Hsp70-c5 -Flag plasmid. **(A)** Digestion by Sif I, lane 1: DNA Marker DL2000, lane 2: pBT3STE-VP1 plasmid digested by restriction endonuclease. **(B)** Digestion by *Nco*I and *Hin*dIII, lane 1: DNA marker DL2000, lane 2:pET32a-Hsp70-c5 plasmid digested by restriction endonuclease. **(C)** Digestion by EcorI and *Hin*dIII, lane 1: DNA marker DL2000, lane 2:pTT5-Hsp70-c5 plasmid digested by restriction endonuclease. **(D)** Digestion by EcorI and *Kpn*I, lane 1: DNA marker DL2000, lane 2:pEGFP-C2-Hsp70-c5 plasmid digested by restriction endonuclease.

After RT-PCR using a specific primer, a fragment of approximately 945 bp was generated (Figure 3A). Sequencing and alignment results revealed that the nucleotide sequence identity exceeded 99% similarity with the reference strain. Then, the *VP1* gene was cloned into the plasmid pBT3STE using the Sif I restriction site. Restriction digestion and sequencing analysis indicated that pBT3STE-VP1 bait plasmid was successfully constructed.

### Function and Self-Activation Detection of pBT3STE-VP1 Plasmid

**FIGURE 4:**
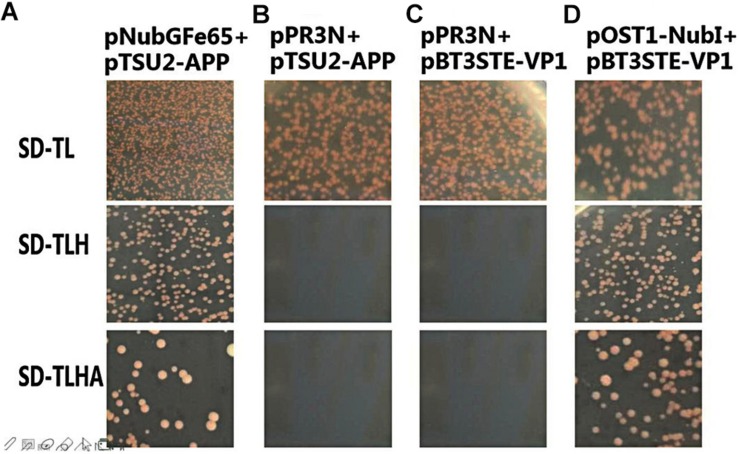
Function and self-activation detection of pBT3STE-VP1 plasmid. Panel **(A)** is the positive control group: pNubGFe65 and pTSU2-APP. Panel **(B)** is the Negative control group: pPR3N and pTSU2-APP. Panel **(C)** is the self-activation assay of the bait plasmid pBT3STE-VP1: pPR3N and pBT3STE-VP1. Panel **(D)** is the functional test group: pOST1-NubI and pBT3STE-VP1.

To analyze the self-activation level of the pBT3STE-VP1 bait plasmid in yeast cells, pBT3STE-VP1 and various plasmids were co-transfected into NMY32 cells and assayed on selective plates. Analysis of the function and self-activation of *VP1* revealed that white clones grew on all the SD/-Trp/-Leu plates. White clones were present on the SD/-Trp/-Leu/-His and SD/-Trp/-Leu/-Ade/-His solid plates in the positive control and functional test groups but were absent in the negative control and self-activation detection groups (Figure 4). Therefore, the pBT3STE-VP1 bait plasmid had functions and exhibited no self-activation in the assay.

### *Apis cerana* Larvae cDNA Library Screening Using pBT3STE-VP1 as the Bait

To screen host proteins that interplay with the pBT3STE-VP1 bait using the Y2H system, the bait plasmid pBT3STE-VP1 was hybridized using the cDNA library of *A. cerana*. The clones were screened, yielding 40 positive bait–prey interacting yeast from the membrane protein Y2H system, and passed the detection of reporter genes (Supplementary Figure S1). In addition, sequence analysis of the positive plasmids indicated that these 40 plasmids represented 23 cDNA of potential protein genes interacting with *VP1* (Supplementary Table S1). The gene screened was *Hsp70* gene (GenBank access No. MH122655.1, named *Hsp70-c5*) which identity with the *A. cerana* heat shock 70 kDa protein cognate 5 (GenBank access No. XM017048256.1) is 83.35%.

### His and *Hsp70-c5* Fusion Protein Expression and Purification From *E. coli*

**FIGURE 5:**
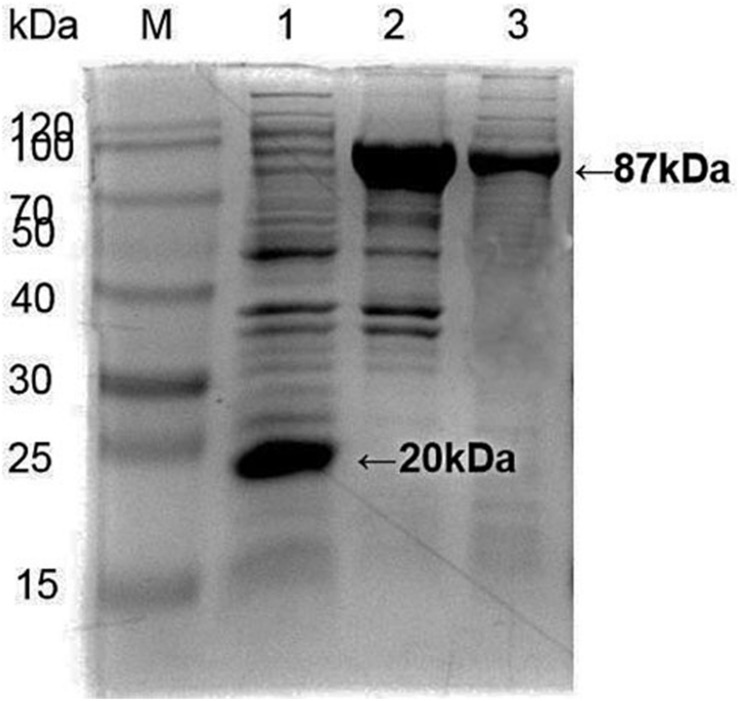
Expression and purification of *E. coli* HIS and *Hsp70-c5* Fusion protein. Purification of Recombinant protein pET-32a-Hsp70-c5, M: the low molecular weight standard protein Marker, lane 1: Expression of pET-32a, lane 2: Expression of HIS-Hsp70-c5, lane 3: pET-32a-Hsp70-c5 purification.

*Apis cerana Hsp70-c5* complete cDNA had an overall length of 1833 bp. Recombinant plasmids pET32a-Hsp70-c5 were constructed using *Nco*I and *Hin*dIII (Figure 3B). The expression plasmids were transformed into BL21 (DE3). After induction, His-Hsp70-c5 recombinant protein could be purified effectively using the His-tagged Protein Purification Kit. Protein expression levels were analyzed using SDS-PAGE. The results indicated that the recombinant proteins had molecular weights of 87 kDa. However, the HIS protein in the negative control had a molecular weight of approximately 20 kDa (Figure 5).

### GST Pull-Down Assay

**FIGURE 6:**
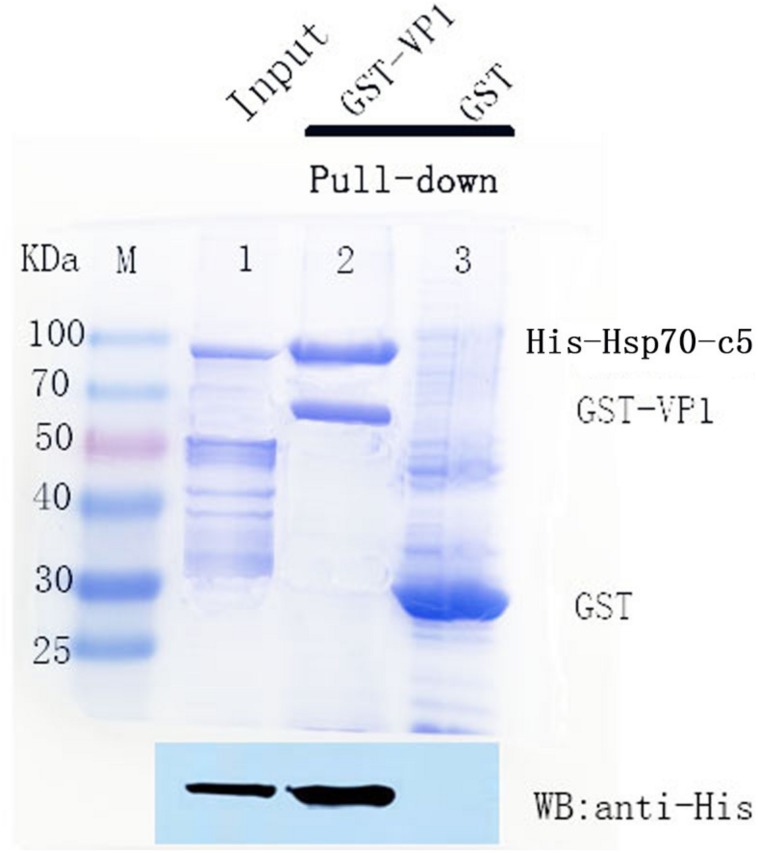
SDS-PAGE (8%, Coomassie blue stained) and western blotting analysis of glutathione S-transferase (GST) pull-down samples. Compared with the negative control group, the experimental and positive control groups exhibited a protein band with a molecular weight of approximately 87 kDa, consistent with the expected *Hsp70-c5* protein sizes **(Upper)**, indicating that *Hsp70-c5* and GST-VP1 may have formed a complex. Western blotting was performed with anti-His antibody. His-Hsp70-c5 protein appeared at a molecular mass of approximately 87 kDa **(Lower)**. GST-VP1 pull down Hsp70-c5-His. SDS-PAGE analysis (Coomassie blue-stained) of GST-VP1 pull down samples **(Upper)**, and western blotting was performed using anti-His antibody **(Lower)**.

To detect the binding ability between *VP1* and host *Hsp70-c5 in vitro*, GST pull-down assay was performed with GST, GST-VP1, and *Hsp70-c5*. The experimental results were analyzed using Coomassie blue staining and western blotting (Figure 6). Compared with the negative control group, the experimental and positive control groups exhibited a protein band with a molecular weight of approximately 87 kDa, consistent with the expected *Hsp70-c5* protein sizes (Upper), indicating that *Hsp70-c5* and GST-VP1 may have formed a complex. Western blotting was performed with anti-His antibody. His-Hsp70-c5 protein appeared at a molecular mass of approximately 87 kDa (Lower). The results indicate that there is a strong interaction between *VP1* and *Hsp70-c5*.

### Co-IP Assay

**FIGURE 7:**
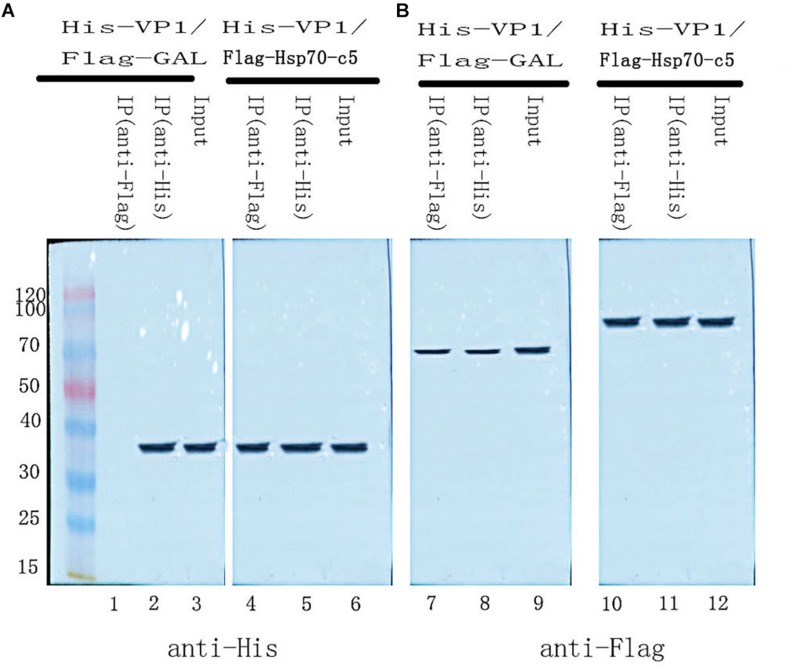
Used Co-IP assay analysis interaction between *VP1* and *Hsp70-c5.* Immunoprecipitation of *VP1* and *Hsp70-c5.* HEK293T cells were co-transfected with the expression plasmids for His-tagged *VP1* and FLAG-tagged *Hsp70-c5.* The resulting Input and IP samples (anti-His or anti-Flag) were subjected to SDS-page electrophoresis, and protein expression was detected using His and Flag antibodies. **(A)** The resulting Input and IP samples (anti-His or anti-Flag) were subjected to SDS-page electrophoresis, and protein expression was detected using His antibodies. **(B)** The resulting Input and IP samples (anti-His or anti-Flag) were subjected to SDS-page electrophoresis, and protein expression was detected using Flag antibodies.

To further determine whether the interaction between *VP1* and *Hsp70-c5* occurs *in vitro*, Co-IP was performed in HEK293T cells co-expressing Hsp70-c5-FLAG and VP1-His. Recombinant plasmid pTT5-Hsp70-c5-Flag was constructed using *Eco*RI and *Hin*dIII (Figure 3C). Co-IP assay was performed in HEK293T cells co-expressing Hsp70-c5-Flag and VP1-His. The results show that Hsp70-c5-Flag could be co-immunoprecipitated with anti-His antibodies or anti-Flag when co-expressed with VP1-His, demonstrating an interaction between Hsp70-c5-Flag and VP1-His in the cells (Figure 7).

### Immunofluorescence Staining and Confocal Microscopy

**FIGURE 8:**
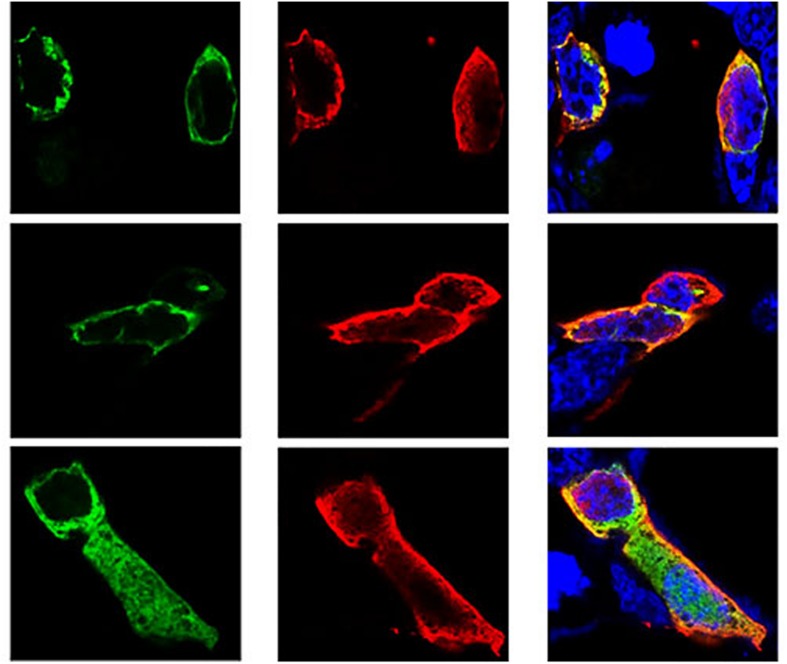
Colocalization of *VP1* and *Hsp70-c5* proteins using immunofluorescence confocal. Green represents Flag-Hsp70-c5, red represents His-VP1, blue DAPI represents nucleus, co-localization shows that red is almost completely coincident with green. Colocalization of *VP1* and *Hsp70-c5* is indicated in yellow in this merged.

Following the determination of interactions between *Hsp70-c5* and *VP1*, we investigated whether these two proteins colocalized in the cells. To determine whether *VP1* and *Hsp70-c5* proteins localized within the same cellular compartment, recombinant pEGFP-C2-Hsp70-c5-Flag plasmid was constructed using *Eco*RI and *Kpn*I restriction sites (Figure 3D). The recombinant plasmids pTT5-VP1-His and pEGFP-C2-Hsp70-c5-Flag were co-transfected into HEK293T cells. As shown by the green fluorescent dots in Figure 8, Flag-Hsp70-c5 localization in the cytoplasm and nucleus, His-VP1 also localization in the cytoplasm and nucleus. *VP1* and *Hsp70-c5* colocalized in an ER-like pattern, which was particularly evident in the cytoplasm and nucleus. Colocalization results showed that co-expressed *VP1* and *Hsp70-c5* could completely overlap in cells. Our data illustrate that *VP1* and *Hsp70-c5* were colocalized primarily in cytoplasm (Figure 8).

### *Hsp70-c5* Level After Larvae Infected With CSBV

**FIGURE 9:**
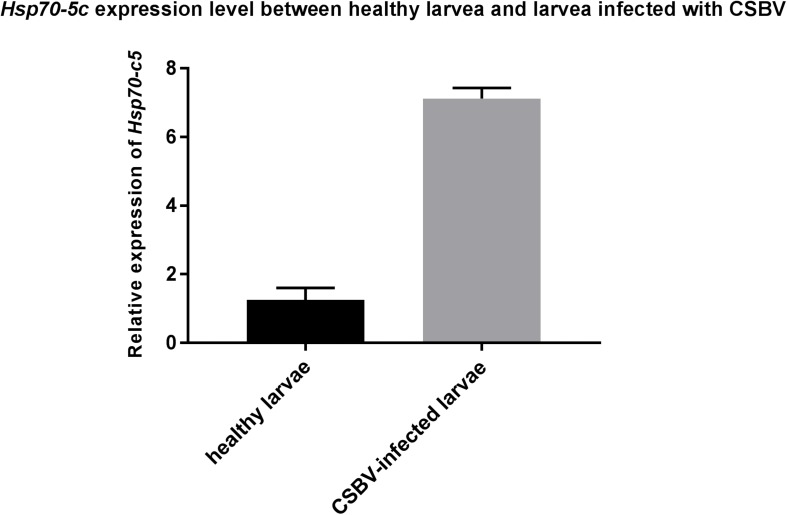
*Hsp70-c5* level after larvae infected with CSBV. *Hsp70-c5* level after larvae infected with CSBV. The bars represent relative fold change of *Hsp70-c5* expression. The black bars indicates expression of *Hsp70-c5* in the larvae, and gray bars denote higher expression of *Hsp70-c5* in the infected larvae. Error bar is standard deviation.

These experiments were performed to further investigate the changes in *Hsp70-c5* expression in larvae after CSBV infection. The expression levels of *Hsp70-c5* in each group of larvae were detected via SYBR Green RT-PCR. *Hsp70-c5* expression in larvae was detected using TransStart Tip Green qPCR SuperMix (TransGen, Beijing, China), and the copy number was calculated according to the method of Zhou et al. (2017). The results indicated that *Hsp70-c5* was expressed in honeybee larvae. *Hsp70-c5* expression levels were low in healthy larvae, whereas it was high in the CSBV-infected larvae (Figure 9). All dead larvae found during the experiments were analyzed by RT-PCR, and the results showed that all of the other honeybee viruses were undetectable, whereas CSBV was detectable.

**FIGURE 10:**
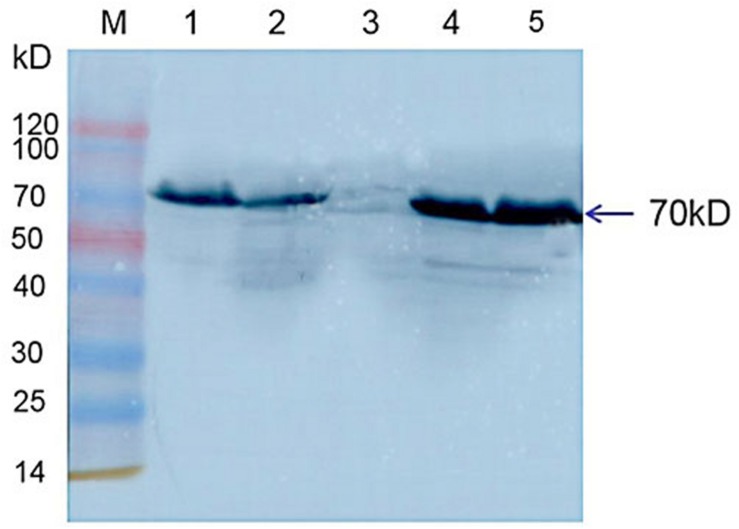
Detection of Hsp70-c5 expression in three groups of healthy and diseased larvae. M: Low molecular weight standard protein Marker; 1, 2: The expression of *Hsp70-c5* in healthy larvae; 4, 5: Expression of *Hsp70-c5* in the infected larva of CSBV.

Western blot results indicated that *Hsp70-c5* was expressed in honeybee larvae and its molecular weight was approximately 70 kDa. *Hsp70-c5* gene expression levels were low in healthy larvae, whereas they were high in the CSBV-infected larvae (Figure 10). The results indicated that *Hsp70-c5* expression considerably increased in CSBV-infected larvae. Therefore, we conclude that *Hsp70-c5* upregulation can affect viral assembly processes or enhance host antiviral effects.

### Effect of Silencing *Hsp70-c5* on *VP1* Expression

We have founded that *Hsp70-c5* expression was upregulated *in vivo* when the larvae were infected with CSBV. We performed silencing experiments to investigate the function of *Hsp70-c5* during CSBV infection more specifically. Compared with siRNA1 and siRNA2, siRNA3 had the highest inhibitory effect on *Hsp70-c5*. Therefore, siRNA3 was chosen for the subsequent experiment. Comparing the expression levels of *Hsp70-c5* in groups V and VII, we found that *Hsp70-c5* expression was downregulated in groups V. Comparing the expression levels of *VP1* in groups V and VI, we found that when we silenced *Hsp70-c5, VP1* expression was significantly downregulated, indicating that *Hsp70-c5* can promote the replication of CSBV in larvae.

## Discussion

The honeybee is an important insect species for crop pollination and plays a crucial role in agricultural production (Potts et al., 2011). CSBV is the pathogen that majorly infects *A. cerana*, resulting in severe and lethal infections in colonies and eventual losses of entire colonies (Feng et al., 2013). *VP1* reportedly has the highest variation in amino acid sequences among different CSBV strains. In addition, it harbors the major CSBV epitope (Mingxiao et al., 2011). Cheng et al. (2011) demonstrated that *VP1* is an excellent immunogen in CSBV and structural proteins are a major component of the virus, although the role of *VP1* in viral infection and its interaction with host proteins remains unclear. Studies on the interaction between *VP1* and host proteins are important and could improve our understanding of the molecular mechanisms underlying viral proliferation and pathogenesis and enable the identification of checkpoints for the development of appropriate therapeutics.

Chinese sacbrood virus is a typical positive single-stranded RNA virus. RNA viruses often rely on host factors to proliferate in cells (Snijder, 2008; Tang et al., 2012; Reid et al., 2015); therefore, we screened out the host proteins interacting with CSBV *VP1* using the membrane protein Y2H system in the present study. According to the results, 23 host proteins that interacted with *VP1* were identified. The interacting proteins were associated with critical biological functions, such as stress response, protein phosphorylation, signal transduction, protein targeting, cytoskeleton organization, DNA repair, protein regulation, ribosome metabolism. Among these interacting proteins, *Hsp70* is majorly significant in research owing to its roles in viral proliferation and immunity (Kantengwa et al., 1995; Carsillo et al., 2009).

*HSPs* are widely expressed in prokaryotes and eukaryotes as stress-inducible molecular chaperons (Shukla and Pitha, 2012). Based on the molecular weights of *HSPs*, they can be classified as *Hsp100*, *Hsp90*, *Hsp70*, *Hsp60*, *Hsp40*, small *Hsp* family, and ubiquitin, with *Hsp70* being the most widely studied (Venetianer et al., 1994). Zhang (2013) reported that there are at least five *Hsp70s* in honeybee, such as *Hsp70Ab, Hsc70-4, Hsc70-3, Hsc70-5, Hsp70-4L*. *Hsp70* is involved in immune responses and participates in the life of a virus through folding, transporting, positioning, assembling, or degrading activities (Risler et al., 2012). Increasing evidence suggests that *Hsp70* plays key roles in viral replication and assembly in several RNA viruses (Nagy et al., 2011; Lahaye et al., 2012; Katoh et al., 2017), such as those of dengue (Taguwa et al., 2015), avian infectious bronchitis (Shan et al., 2018), and influenza A (Manzoor et al., 2014). Alzhanova et al. (2014) observed that *Hsp70* could bind to the structural proteins of beet yellows virus to form a complex and participate in viral assembly. In addition, Jiang et al. (2015) demonstrated that *Hsp70* probably participates in rice stripe viral replication by interacting with viral RNA-dependent RNA polymerase (*RdRp*). In the present study, utilizing the GST pull-down and Co-IP assays, we have revealed an interaction that exists between structural protein *VP1* and *Hsp70-c5*. In addition, we speculate that the interaction between *VP1* and *Hsp70-c5* in *A. cerana* plays a role in viral assembly.

Confocal microscopy was used to investigate the colocalization of *Hsp70-c5* and *VP1* in HEK293T cells. *VP1* and *Hsp70-c*5 were mainly colocalized in the nucleus and cytoplasm. Ren et al. (2012) demonstrated that *Hsp70* migrated into the raft fraction after Japanese encephalitis virus infection, whereas Ye et al. (2001) observed that in Hantaan virus infection, *Hsp70* forms a complex with nuclear proteins, which translocates from the cytoplasm to nucleoli, a process associated with viral assembly. *Hsp70* influences viral assembly and enhances host antiviral immunity in the course of viral infection (Kim et al., 2013b). Ohgitani et al. (1999) analyzed the movement of *Hsp70* from the cytoplasm to the nucleus and then back to the cytoplasm at different stages of infection and observed that in the early stages of infection, due to the presence of stress factors, *Hsp70* aggregates in the nucleus and may be involved in the transcription processes of viruses and hosts, viral DNA replication, and assembly of nucleocapsid proteins. However, *Hsp70* aggregates in the cytoplasm and is involved in the protection of infected cells from host immunity in the later stages of infection. Therefore, we speculated that *Hsp70-c5* migrates during the course of CSBV infection, a process that potentially influences viral assembly and immune processes.

*Hsp70* upregulation after viral infection has been widely reported (Padwad et al., 2010). Lahaye et al. (2012) observed that *Hsp70* upregulation was correlated with excess amounts of viral proteins, suggesting that *Hsp70* plays an active role in the rabies viral life cycle. Vesicular stomatitis viral infection results in *Hsp70* upregulation, which ultimately induces innate immune responses that facilitate viral clearance (Kim et al., 2013a). In the present study, *Hsp70-c5* expression in CSBV-infected larvae was significantly upregulated when compared with healthy larvae. Thus, we conclude that *Hsp70-c5* upregulation can affect viral assembly processes or enhance host antiviral effects.

However, further studies are required to determine whether the interactions between *Hsp70-c5* and *VP1* play a role in CSBV infection. De-la-Re-Vega et al. (2017) observed that *Hsp70* is involved in the process of herpes virus infection, thereby promoting herpes virus replication. Taguwa et al. (2015) found that when dengue virus infects the body, *Hsp70* is involved in every step of viral infection, i.e., from replication to assembly to release, to help the virus complete the infection. Han et al. (2013) also found *Hsc70* and *Hsp90* participate in the facilitation of viral production in host cells during the infection cycle. In the larval experiment, we found that HSP70 expression was upregulated *in vivo* when the larvae were infected with CSBV. We performed silencing experiments to investigate the function of *Hsp70-c5* during CSBV infection more specifically and found that when we silenced *Hsp70-c5*, VP1 expression was significantly downregulated, indicating that *Hsp70-c5* can promote the replication of CSBV in larvae. These results demonstrate that *Hsp70-c5* is involved in at least one stage of the viral life cycle.

In summary, we screened out the host protein, *Hsp70-c5*, as an interacting partner with *VP1* of CSBV using the membrane protein Y2H system and observed the interaction between *VP1* and *Hsp70-*c5 using the GST pull-down and Co-IP assays. *VP1* and *Hsp70-*c5 were colocalized mainly in the nucleus and cytoplasm and *Hsp70-c5* expression in CSBV-infected larvae was significantly upregulated. Our study supports a positive regulatory role for *Hsp70-c5* in the CSBV virus infection cycle. Our findings could facilitate the study of the mechanisms underlying CSBV infection and host antiviral defense. The host–pathogen interaction reported in the present study could serve as a foundation for the development of novel therapeutics and prevention strategies against CSBV infections.

## Data Availability Statement

The datasets generated for this study are available on request to the corresponding author.

## Ethics Statement

The use of the experimental animals involved in the manuscript is in compliance with the relevant provisions of the Animal Welfare and Ethics of Experimental Animals of the Experimental Animal Center of Jinzhou Medical University, China.

## Author Contributions

XZ and DF designed the study and wrote the manuscript. LS, ML, DF, SH, CW, YM, and MM performed the experiments and analyzed the data. All authors approved the final version of the manuscript.

## Conflict of Interest

The authors declare that the research was conducted in the absence of any commercial or financial relationships that could be construed as a potential conflict of interest.

## Supplementary Material

FIGURE S1 Screening of proteins interacting with pBT3STE-VP1 plasmid and yeast Library Gel electrophoresis of PCR products amplified from putatively positive prey plasmids.

TABLE S1 Analysis of sequencing results.
