# Supplementary figures and images for: Identification of the Novel Host Protein Interacting With the Structural Protein VP1 of Chinese Sacbrood Virus by Yeast Two-Hybrid Screening
Source: Front Microbiol. 2019 Sep 26;10:2192. doi: 10.3389/fmicb.2019.02192 (PMC6775477; doi:10.3389/fmicb.2019.02192)

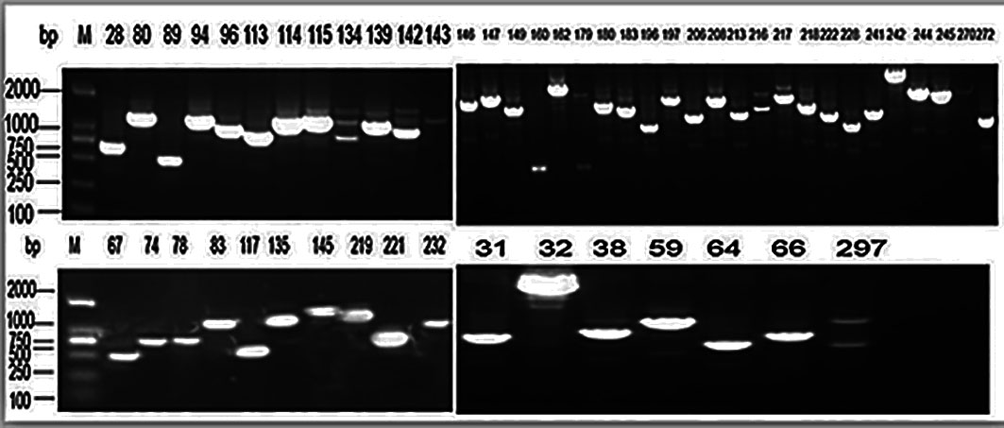

Supplement: FIGURE S1 — Screening of proteins interacting with pBT3STE-VP1 plasmid and yeast Library Gel electrophoresis of PCR products amplified from putatively positive prey plasmids. [file Image_1.TIF]
