# Supplementary material for: Identification of the Novel Host Protein Interacting With the Structural Protein VP1 of Chinese Sacbrood Virus by Yeast Two-Hybrid Screening
Source: Front Microbiol. 2019 Sep 26;10:2192. doi: 10.3389/fmicb.2019.02192 (PMC6775477; doi:10.3389/fmicb.2019.02192)
Supplement: TABLE S1 — Analysis of sequencing results. [file Table_1.DOC]

Supplementary Table 1 Analysis of sequencing results

| Positive clone number | Sequence BLAST alignment analysis | |
| --- | --- | --- |
|  | Gen Bank | English name |
| 113 | [XM_006618695.1](https://www.ncbi.nlm.nih.gov/nucleotide/XM_006618695.1?report=genbank&log$=nucltop&blast_rank=2&RID=D7VTXUK301R) | Apis mellifera sex-regulated protein |
| 59, 114, 228 | [XM_017054579.1](https://www.ncbi.nlm.nih.gov/nucleotide/XM_017054579.1?report=genbank&log$=nucltop&blast_rank=1&RID=D8KWVEBB014)  [XM_017054579.1](https://www.ncbi.nlm.nih.gov/nucleotide/XM_017054579.1?report=genbank&log$=nucltop&blast_rank=1&RID=D8KWVEBB014)  [XM_393059.7](https://www.ncbi.nlm.nih.gov/nucleotide/XM_393059.7?report=genbank&log$=nucltop&blast_rank=1&RID=D8M5RJNS015) | Apis cerana 40S ribosomal protein |
| 115 | [XM_017055329.1](https://www.ncbi.nlm.nih.gov/nucleotide/XM_017055329.1?report=genbank&log$=nucltop&blast_rank=1&RID=D8M9BU9Y015) | Apis cerana proteasomal ubiquitin receptor |
| 139 | [XM_017048672.1](https://www.ncbi.nlm.nih.gov/nucleotide/XM_017048672.1?report=genbank&log$=nucltop&blast_rank=1&RID=D8MC774S015) | Apis cerana peroxiredoxin |
| 28, 31, 94, 117, 196, 206 | [XM_017065619.1](https://www.ncbi.nlm.nih.gov/nucleotide/XM_017065619.1?report=genbank&log$=nucltop&blast_rank=1&RID=D8MM8FEN014)  [XM_017050055.1](https://www.ncbi.nlm.nih.gov/nucleotide/XM_017050055.1?report=genbank&log$=nucltop&blast_rank=1&RID=D8MV5CFJ014)  [XM_017063945.1](https://www.ncbi.nlm.nih.gov/nucleotide/XM_017063945.1?report=genbank&log$=nucltop&blast_rank=1&RID=D8MWB62E014)  [XM_017063945.1](https://www.ncbi.nlm.nih.gov/nucleotide/XM_017063945.1?report=genbank&log$=nucltop&blast_rank=1&RID=D8MWB62E014)  [XM_001120364.5](https://www.ncbi.nlm.nih.gov/nucleotide/XM_001120364.5?report=genbank&log$=nucltop&blast_rank=1&RID=D8N610JH01R)  [XM_017066291.1](https://www.ncbi.nlm.nih.gov/nucleotide/XM_017066291.1?report=genbank&log$=nucltop&blast_rank=1&RID=D8NBXPSU014) | Apis cerana 60S ribosomal protein |
| 43, 142 | [XM_017050521.1](https://www.ncbi.nlm.nih.gov/nucleotide/XM_017050521.1?report=genbank&log$=nucltop&blast_rank=1&RID=D8NY5YA2015)  XM_017050521.1 | Apis cerana NADH dehydrogenase [ubiquinone] iron-sulfur protein |
| 80 | [XM_017049780.1](https://www.ncbi.nlm.nih.gov/nucleotide/XM_017049780.1?report=genbank&log$=nucltop&blast_rank=1&RID=D8PGFCN6014) | Apis cerana balbiani ring protein |
| 96, 134, 222, 272 | [KM244704.1](https://www.ncbi.nlm.nih.gov/nucleotide/KM244704.1?report=genbank&log$=nucltop&blast_rank=1&RID=D8PKPY0A014)  [KM244704.1](https://www.ncbi.nlm.nih.gov/nucleotide/KM244704.1?report=genbank&log$=nucltop&blast_rank=1&RID=D8PKPY0A014)  [KM244704.1](https://www.ncbi.nlm.nih.gov/nucleotide/KM244704.1?report=genbank&log$=nucltop&blast_rank=1&RID=D8PW6YC1014)  [KM244704.1](https://www.ncbi.nlm.nih.gov/nucleotide/KM244704.1?report=genbank&log$=nucltop&blast_rank=3&RID=D8PZHRD3014) | Apis cerana isolate CL24 mitochondrion |
| 135 | [XM_017061853.1](https://www.ncbi.nlm.nih.gov/nucleotide/XM_017061853.1?report=genbank&log$=nucltop&blast_rank=1&RID=D8R4PTDY014) | Apis cerana methionine-tRNA ligase |
| 145, 146 | [XM_006624424.1](https://www.ncbi.nlm.nih.gov/nucleotide/XM_006624424.1?report=genbank&log$=nucltop&blast_rank=1&RID=D8R724DJ014)  [XM_006624424.1](https://www.ncbi.nlm.nih.gov/nucleotide/XM_006624424.1?report=genbank&log$=nucltop&blast_rank=1&RID=D8R8NCYJ014) | Apis dorsata 3-hydroxyacyl-CoA |
| 149 | [XM_017059853.1](https://www.ncbi.nlm.nih.gov/nucleotide/XM_017059853.1?report=genbank&log$=nuclalign&blast_rank=1&RID=D8RAZG55014) | Apis cerana protein G12 |
| 162 | [XM_006616514.1](https://www.ncbi.nlm.nih.gov/nucleotide/XM_006616514.1?report=genbank&log$=nucltop&blast_rank=1&RID=D8RG2G7T014) | Apis dorsata scaffold protein |
| 180 | [XM_017058135.1](https://www.ncbi.nlm.nih.gov/nucleotide/XM_017058135.1?report=genbank&log$=nuclalign&blast_rank=1&RID=D8RHTY2C014) | Apis cerana cullin-2 |
| 208 | [XM_017048256.1](https://www.ncbi.nlm.nih.gov/nucleotide/XM_017048256.1?report=genbank&log$=nuclalign&blast_rank=1&RID=D8RMTSN5014) | Apis cerana heat shock 70 kDa protein |
| 213 | [XM_017052341.1](https://www.ncbi.nlm.nih.gov/nucleotide/XM_017052341.1?report=genbank&log$=nucltop&blast_rank=1&RID=D8RPTU6R014) | Apis cerana coiled-coil-helix-coiled-coil-helix domain-containing protein |
| 66, 217, 219 | [XM_017054216.1](https://www.ncbi.nlm.nih.gov/nucleotide/XM_017054216.1?report=genbank&log$=nuclalign&blast_rank=1&RID=D8RUD5DH014)  [XM_017054216.1](https://www.ncbi.nlm.nih.gov/nucleotide/XM_017054216.1?report=genbank&log$=nuclalign&blast_rank=1&RID=D8RXW551014)  [XM_017054216.1](https://www.ncbi.nlm.nih.gov/nucleotide/XM_017054216.1?report=genbank&log$=nuclalign&blast_rank=1&RID=D8S0KJ5W014) | Apis cerana aldose reductase |
| 221 | [XM_017056670.1](https://www.ncbi.nlm.nih.gov/nucleotide/XM_017056670.1?report=genbank&log$=nuclalign&blast_rank=1&RID=D8S2GXCU014) | Apis cerana leucine-rich repeat-containing protein |
| 241 | [XM_006624129.1](https://www.ncbi.nlm.nih.gov/nucleotide/XM_006624129.1?report=genbank&log$=nucltop&blast_rank=1&RID=D8S6F930014) | Apis dorsata baculoviral IAP repeat-containing protein |
| 244 | [XM_017059135.1](https://www.ncbi.nlm.nih.gov/nucleotide/XM_017059135.1?report=genbank&log$=nuclalign&blast_rank=1&RID=D8S9G2YC015) | Apis cerana heat shock 83 kDa protein |
| 38 | [XM_017056283.1](https://www.ncbi.nlm.nih.gov/nucleotide/XM_017056283.1?report=genbank&log$=nuclalign&blast_rank=1&RID=D8SAZ0M8014) | Apis cerana epididymal secretory protein |
| 64 | [XM_017063537.1](https://www.ncbi.nlm.nih.gov/nucleotide/XM_017063537.1?report=genbank&log$=nuclalign&blast_rank=1&RID=D8SC6HSK014) | Apis cerana peritrophin |
| 74 | [XM_017060151.1](https://www.ncbi.nlm.nih.gov/nucleotide/XM_017060151.1?report=genbank&log$=nuclalign&blast_rank=1&RID=D8SE2P4J014) | Apis cerana eukaryotic initiation factor |
| 78, 147, 197, 218 | [XM_017060011.1](https://www.ncbi.nlm.nih.gov/nucleotide/XM_017060011.1?report=genbank&log$=nuclalign&blast_rank=1&RID=D8SGR2Y9014)  [XM_017056086.1](https://www.ncbi.nlm.nih.gov/nucleotide/XM_017056086.1?report=genbank&log$=nucltop&blast_rank=1&RID=D8SHSMSK014)  [XM_017058024.1](https://www.ncbi.nlm.nih.gov/nucleotide/XM_017058024.1?report=genbank&log$=nucltop&blast_rank=1&RID=D8SK8VPV01R)  [XM_017066549.1](https://www.ncbi.nlm.nih.gov/nucleotide/XM_017066549.1?report=genbank&log$=nucltop&blast_rank=1&RID=D8SNFZX901R) | Apis cerana uncharacterized |
